# Substance use disorder and alcohol consumption patterns among Dutch physicians: a nationwide register-based study

**DOI:** 10.1186/s13722-022-00356-9

**Published:** 2023-01-13

**Authors:** Pauline Geuijen, Arnt Schellekens, Aart Schene, Femke Atsma

**Affiliations:** 1 Donders Institute for Brain, Cognition and Behaviour, Centre for Neuroscience, Department of Psychiatry, Radboud University Medical Center, Reinier Postlaan 4, 6525 GC Nijmegen, The Netherlands; 2 Nijmegen Institute for Scientist-Practitioners in Addiction (NISPA), Nijmegen, The Netherlands; 3 Scientific Center for Quality of Healthcare (IQ Healthcare), Radboud University Medical Center, Radboud Institute for Health Sciences, Nijmegen, The Netherlands

**Keywords:** Physicians, Highly educated, Diagnoses, Substance use disorder, Alcohol consumption patterns

## Abstract

**Purpose:**

Problematic substance use and Substance Use Disorders (SUD) are common in all layers of the population. Several studies suggest higher prevalence rates of problematic substance use among physicians compared to the general population, which is harmful for themselves and potentially impairs quality of care. However, nationwide comparison with a highly educated reference group is lacking. Using nationwide register data, this study compared the prevalence of clinical SUD diagnoses and alcohol consumption patterns between physicians and a highly educated reference population.

**Methods:**

A retrospective study was performed using registry data from 2011 up to and including 2019, provided by Statistics Netherlands. From the data, a highly educated reference group was selected and those with an active medical doctor registration were identified as “physicians”. Clinical SUD diagnoses were identified by DSM-IV codes in mental healthcare registries. Benchmark analyses were performed, without statistical testing, to compare the prevalence of SUD diagnoses and alcohol consumption patterns between physicians and the reference population.

**Results:**

Clinical SUD diagnoses were found among 0.3% of the physicians and 0.5% of the reference population, with higher proportions of sedative use disorder among physician patients. Among drinkers, the prevalence rates of heavy and excessive drinking were respectively 4.0% and 4.3% for physicians and 7.7% and 6.4% for the reference population.

**Conclusion:**

Prevalence rates of SUD diagnoses were fairly comparable between physicians and the highly educated reference population, but physicians displayed more favorable alcohol consumption patterns. The use of sedatives by physicians might deserve attention, given the relatively higher prevalence of sedative use disorder among physicians. Overall, we observed relatively low prevalence rates of SUD diagnoses and problematic alcohol use, which may reflect a treatment gap and social desirable answers.

**Supplementary Information:**

The online version contains supplementary material available at 10.1186/s13722-022-00356-9.

## Introduction

Substance use disorders (SUD) are a common disorder, affecting all layers of the population. SUDs are associated with personal harm and impaired general functioning [17]. For several professions the impairing effects of SUD are of particular societal relevance. For instance, in pilots and physicians SUD-induced impairment can have tremendous consequences for others dependent on the quality of their work for their safety. Consequently, specific care programs have been developed for such professionals, including Physician Health Programs (PHPs) [3, 4, 11]. Since 2011, the Royal Dutch Medical Association (RDMA) offers a Physician Health Program (PHP) for physicians with SUD [10]. This PHP guarantees confidentiality by not having any formal links with regulating authorities. Conversely, the regulator does refer a physician with SUD to the Dutch PHP.

While data from the United States suggests lower prevalence rates of SUD among physicians compared to the general population, these data are hampered by several methodological shortcomings, including variation in assessment between groups. The self-reported lifetime prevalence of SUD in a large sample of physicians (n = 5,426) (8%), was much lower than the SUD prevalence in the general population (16%), which was estimated by diagnostic interviews [15, 23]. In addition, higher socioeconomic status (SES) among physicians compared to the general population might be a confounding factor [15]. In contrast, European data suggested that problematic alcohol use was higher among physicians (13–30%) compared to the general population (7–15%) [16, 26, 36, 41], though again different measures were used for both groups.

So far, only two small scale observational studies among physicians (n = 99) and healthcare professionals (n = 94) addressed a decent comparison to a reference population by including an educational status-matched community sample of non-physicians (n = 99) and a clinical sample of highly educated non-healthcare professionals (n = 45) [8, 24]. Physicians and healthcare professionals showed significantly higher odds of SUD of opioids and sedatives, compared to the control group [8, 24].

Taken together, inconclusiveness exists about the SUD prevalence among physicians and how this prevalence relates to the one in the general population. On the one hand, physicians might be more at risk for developing SUD due to an extensive work load, irregular working hours, and easy access to prescription drugs [5, 27], and on the other hand, physicians might be at lower risk for SUD because of their socioeconomic status (SES; high level of education, high income, and favorable position on the labor market) [34, 37]. Due to the potential negative impact of SUD in physicians on their functioning and patient safety, it is important to get insight in prevalence rates of SUD among physicians and to establish the magnitude of the problem. Additionally, prevalence rates provide insight into the existence of an occupational risk for SUD among physicians. In case of such an occupational risk, this would give rise to targeted prevention and perhaps preventive monitoring among physicians.

In the current study, we used nationwide register data provided by Statistics Netherlands to explore whether physicians might be more at risk for developing SUD due to work related factors. We selected a reference population of Dutch citizens with an educational level comparable to that of physicians. A comparison between the physicians and the highly educated reference population was made with respect to the prevalence of clinical SUD diagnoses and alcohol consumption patterns as well as psychiatric and somatic comorbidity, general functioning, and sociodemographic characteristics.

## Methods

### Data source

A retrospective study was performed using data, provided by Statistics Netherlands. We selected data about highly educated Dutch citizens, physician registrations, clinical SUD diagnoses, psychiatric and somatic comorbidity, general functioning, alcohol consumption patterns, and sociodemographic characteristics. These data were available from five different registers:

1. Demographics register containing demographics (gender, year of birth, country of birth, educational level, and educational direction) of all legally residing citizens of The Netherlands from 2011 up to and including 2019 (Statistics Netherlands [30, 32]. Statistics Netherlands derives these data from the municipal population registers, educational level registers, and the Labor Force Survey (a rotating panel that is surveyed every quarter).
2. Individual Healthcare Professions register containing data from the Central Information Point for Healthcare Professions [31]. This register includes dates of registration and deregistration, medical profession, and medical specialty.
3. Mental healthcare claims register containing data about diagnoses in Dutch mental healthcare from 1 January 2011 to 31 December 2016 [28, 29]. These diagnoses are based on the Diagnostic and Statistical Manual of Mental Disorders 4th Edition (DSM-IV).
4. Public Health Monitor register containing data on (determinants of) health, social situation, and lifestyle in a sample of Dutch citizens in 2012 and 2016 [6, 7]. The Public Health Monitor is conducted once every four years by Community Health Services, Statistics Netherlands, and the National Institute for Public Health and the Environment.
5. Health Survey register containing data on health, medical contacts, lifestyle, and preventive behavior in a sample of Dutch citizens from 2014 up to and including 2019 [33]. The Health Survey is an annual survey conducted by Statistics Netherlands, which is part of the Dutch Lifestyle Monitor data collection [9].

### Study population

In the Netherlands, a medical graduate receives a Master’s degree and can either start residency directly, work as a physician-not-in-training (temporary supervised clinical work before residency), or start a PhD trajectory, with only a minority obtaining a PhD [22]. Thus, physicians with a registration in the Individual Healthcare Professions register can either have a Master’s degree or a PhD degree. The demographics register was used to select all Dutch citizens aged between 25 and 65 years with a high educational level (Master or PhD degree) in the period from 2011 up to and including 2016. This population was defined as the “reference population”. Citizens with an active registration as physician between 1 January 2011 and 31 December 2016 were identified as “physicians”, based on the Individual Healthcare Professionals register *(Total population)*. In physicians and the reference population, SUD patients were identified based on DSM-IV coding of the Mental healthcare claims register *(SUD patients)*. The same selections were made for the Public Health Monitor and Health Survey registers in the period from 2012 up to and including 2019 to identify drinkers among the reference population and physicians *(Questionnaire respondents)*.

### Sociodemographic characteristics

Available sociodemographic characteristics included gender, age, country of birth, medical specialty, and educational background. The continuous variable age was recoded into a categorical variable (25 to 34 years, 35 to 44 years, 45 to 54 years, and 55 to 65 years) and country of birth was categorized into three categories (The Netherlands, European, and Non-European). For physicians, medical specialties were divided into five categories: (1) general practice; (2) (psycho) social medicine; (3) contemplative somatic medicine; (4) surgical and supportive medicine; and (5) no specialty, see Additional file 1: Table S1 [12]. Educational background was presented in eight categories for the reference population: (1) education; (2) humanities and arts; (3) social sciences, business and law; 4) science, mathematics and computing; (5) engineering, manufacturing and construction; (6) agriculture and veterinary; (7) health and welfare (including medicine); and 8) services.

From the Public Health Monitor and Health Survey registers information was also available on working hours per week (not working or less than 1 h, 1 to 12 h, 12 to 31 h, and 32 or more hours) and household income quintile (1st (lowest income), 2nd, 3rd, 4th, and 5th (highest income) quintile).

### Definition of SUD diagnoses and alcohol consumption patterns

SUD patients and accompanying comorbidity and functioning were identified by a clinical diagnosis of SUD in the Mental healthcare claims register. Substances of abuse or dependence and comorbid psychiatric disorders were identified by DSM-IV codes on substance-related disorders (Additional file 1: Table S2). DSM-IV codes of comorbid somatic disorders were recoded into a dichotomous variable (“complex” + “singular” versus “none”). DSM-IV codes of start and end scores on the Global Assessment of Functioning (GAF) were divided into three categories: (1) persistent danger to major impairment (GAF 0-40); (2) serious to moderate symptoms (GAF 41-60); and (3) mild to no symptoms (GAF 61-100).

Drinkers were identified by the Public Health Monitor and Health Survey registers as those who reported having consumed at least one alcohol unit in the past 12 months [6, 7],Statistics Netherlands et al. [33]. Among drinkers, those compliant with the alcohol consumption recommendation, heavy drinkers, and excessive drinkers were identified. Compliance with the alcohol consumption recommendation was defined as drinking up to maximum one unit of alcohol per day, in line with the recommendation of the Health Council of the Netherlands [6, 7], Statistics Netherlands et al. [33]. Heavy drinking was defined as consuming six (males) or four (females) or more units of alcohol per day at least once a week in the last 6 months, in line with the definition of the public Health Monitor and the Health Survey [6, 7], Statistics Netherlands et al. [33]. Consuming more than 21 (males) or 14 (females) units of alcohol per week was defined as excessive drinking [6, 7],Statistics Netherlands et al. [33]. The groups of heavy and excessive drinkers were not mutually exclusive and therefore we did not present a group of moderate drinkers, which results in row percentages that do not add up to 100%.

### Data analysis

The registry data allowed us to censor clinical SUD diagnoses and alcohol consumption patterns in the reference population and physicians. First, we used descriptive statistics to perform benchmark analyses between the reference population and the physicians with regard to SUD patients. The prevalence of clinical SUD diagnoses was calculated by dividing the number of (reference or physician) citizens with a clinical SUD diagnosis between 2011 and 2016 by the total number of (reference or physician) citizens between 2011 and 2016. Mean years of clinical SUD diagnosis between 2011 and 2016 were calculated by dividing the total number of clinical SUD diagnoses between 2011 and 2016 by the total number of (reference or physician) SUD patients between 2011 and 2016. Next, clinical SUD diagnoses, psychiatric and somatic comorbidity, and general functioning were compared between reference and physician SUD patients.

Second, respondents of the Public Health Monitor and the Health Survey were taken together and representatives from the reference population and the physicians were identified. First, characteristics of the total sample of questionnaire respondents and drinkers were benchmarked. Next, we performed benchmark analyses for the distribution of alcohol consumption patterns (compliance with the alcohol consumption recommendation, heavy drinkers, and excessive drinkers) within drinkers.

We decided not to test group differences statistically, since p-values are very dependent on sample sizes and may lead to misleading conclusions. Due to large numbers very small differences will become statistically significant (p < 0.001), even if these differences are considered as not relevant. In turn relevant differences in small groups may become not significant, even if differences are considered relevant. We therefore argue and recommend to focus on differences in means and proportions instead of statistical significance. Also in other fields, this is a recommended approach to benchmark and analyze large datasets [2, 25].

Small numbers (< 5) are not reported to prevent disclosure of physicians, in some cases the second smallest cell had to be cleared to avoid retracing. Analyses were performed using the Statistical Package for Social Sciences (SPSS), version 25 for Windows (IBM Corporation, Amonk, NY).

## Results

### General sample characteristics

The entire reference population consisted of 810,188 highly educated citizens aged 25 to 65 years, of whom 38,455 (4.7%) had an active registration as physician between 2011 and 2016 (Table 1). The physicians had somewhat higher proportions of females, were slightly older (2.8 years), and were more often born in the Netherlands than the reference population. Among physicians, most common medical specialty groups were general practice (25.1%), no specialty (22.3%), and contemplative somatic medicine (20.5%). Almost half of the reference population completed education in the direction of social sciences, business and law (47.3%), and more than one fifth completed education in the direction of health and welfare (12.6%) or humanities and arts (11.0%).

**Table 1:** Sociodemographic characteristics of total population and SUD patients

	Total population		SUD patients	
	Reference *(n* = *810 188)*	Physicians *(n* = *38 455)*	Reference *(n* = *4 436; 0.5%)*	Physicians *(n* = *133; 0.3%)*
Gender *(n (%))*				
Male	410 783 (50.7)	17 138 (44.6)	2 992 (67.4)	82 (61.7)
Female	399 405 (49.3)	21 317 (55.4)	1 444 (32.6)	51 (38.3)
Age at cohort entry in years *(mean (SD))*	37.7 (10.7)	40.5 (10.1)	42.9 (10.3)	45.2 (10.1)
25–34 *(n (%))*	376 129 (46.4)	13 071 (34.0)	1096 (24.7)	25 (18.8)
35–44 *(n (%))*	227 568 (28.1)	13 024 (33.9)	1425 (32.1)	39 (29.3)
45–54 *(n (%))*	128 731 (15.9)	7 675 (20.0)	1184 (26.7)	36 (27.1)
55–65 *(n (%))*	77 760 (9.6)	4 685 (12.2)	731 (16.5)	33 (24.8)
Country of birth *(n (%))*				
The Netherlands	692 960 (85.5)	34 205 (88.9)	3 734 (84.2)	118 (88.7)
European	32 982 (4.1)	726 (1.9)	204 (4.6)	4 (3.0)
Non-European	84 246 (10.4)	3 524 (9.2)	498 (11.2)	11 (8.3)
Specialty group *(n (%))*				
General practice	NA	9 657 (25.1)	NA	25 (18.8)
(Psycho) social		5 142 (13.4)		33 (24.8)
Contemplative somatic		7 868 (20.5)		14 (10.5)
Surgical and supportive		7225 (18.8)		10 (7.5)
No specialty		8563 (22.3)		51 (38.3)
Educational background *(n (%))*				
Teaching	59 804 (7.4)	NA	315 (7.1)	NA
Humanities and arts	88 877 (11.0)		789 (17.8)	
Social sciences, business and law	382 871 (47.3)		2 170 (48.9)	
Science, mathematics and computing	67 919 (8.4)		350 (7.9)	
Engineering, manufacturing and construction	73 070 (9.0)		267 (6.0)	
Agriculture and veterinary	13 534 (1.7)		46 (1.0)	
Health and welfare (including medicine)	102 420 (12.6)		367 (8.3)	
Services	13 549 (1.7)		91 (2.1)	

*n* number, *NA* Not Applicable, *SD* Standard Deviation, *SUD* Substance Use Disorder

### SUD diagnoses

Our reference population included 4,436 SUD patients (0.5%) and among physicians we observed 133 SUD patients (0.3%) with a clinical SUD diagnosis between 2011 and 2016 (Table 1). Physicians with SUD were more or less comparable to reference SUD patients in terms of demographics. Physician SUD patients were overrepresented in the specialty groups (psycho) social medicine and no specialty. In the reference population, SUD diagnosis was more common among those with an educational background in humanities and arts.

In the period between 2011 and 2016, SUD patients among physicians and in the reference population had on average 1.7 years of a clinical SUD diagnosis (Table 2). Physician patients were more often than reference patients diagnosed with a SUD on sedative, hypnotic, or anxiolytic drugs (16.5% versus 6.8%) and less often diagnosed with a SUD on alcohol and cannabis (67.7% and 10.5% versus 75.7% and 17.6%, respectively). Psychiatric comorbidity and symptom severity at the time of diagnosis were more or less comparable between physician SUD patients and reference SUD patients. At the end of treatment, a somewhat higher proportion of physician patients experienced mild to no symptoms on the Global Assessment of Functioning compared to reference patients (39.8% versus 33.3%).

**Table 2:** Clinical diagnoses of SUD patients

	SUD patients	
	Reference *(n* = *4 436)*	Physicians *(n* = *133)*
Years of clinical SUD diagnosis between 2011 and 2016 *(mean (SD))*	1.7 (1.1)	1.7 (1.1)
Substance of abuse or dependence *(n (%))*		
Alcohol	3 357 (75.7)	90 (67.7)
Amphetamine	112 (2.5)	*
Cannabis	779 (17.6)	14 (10.5)
Cocaine	387 (8.7)	7 (5.3)
Opioid	150 (3.4)	*
Sedative, hypnotic, or anxiolytic	302 (6.8)	22 (16.5)
Other or unknown substance(s)	177 (4.0)	10 (7.5)
Comorbid psychiatric disorder *(n (%))*		
Developmental disorder	372 (8.4)	10 (7.5)
Cognitive disorder	53 (1.2)	*
Psychotic disorder	196 (4.4)	*
Mood disorder	1 177 (26.5)	42 (31.6)
Anxiety disorder	626 (14.1)	15 (11.3)
Somatoform and/or dissociative disorder	91 (2.1)	6 (4.5)
Personality disorder	1 100 (24.8)	37 (27.8)
Other psychiatric disorder	650 (14.7)	15 (11.3)
Somatic comorbidity *(n (%))*		
Complex	350 (7.9)	13 (9.8)
Singular	791 (17.8)	24 (18.0)
Start score Global Assessment of Functioning *(n (%))*		
Constant danger to major impairment (0–40)	391 (8.8)	9 (6.8)
Serious to moderate symptoms (41–60)	3220 (72.6)	95 (71.4)
Mild to no symptoms (61–100)	681 (15.4)	23 (17.3)
End score Global Assessment of Functioning *(n (%))*		
Persistent danger to major impairment (0–40)	334 (7.5)	6 (4.5)
Serious to moderate symptoms (41–60)	2435 (54.9)	66 (49.6)
Mild to no symptoms (61–100)	1476 (33.3)	53 (39.8)

*n* number, *SUD* Substance Use Disorder

^*^small numbers are not reported to prevent disclosure

### Survey sample characteristics (Public Health Monitor and Health Survey)

Our total sample of questionnaire respondents consisted of 32,309 reference citizens (Public Health Monitor n = 29,597; Health Survey n = 2,712) of whom 1,947 (6.0%) were physicians (Public Health Monitor n = 1808; Health Survey n = 139) (Additional file 1: Table S3). Benchmarked to the reference population, physician respondents showed higher proportions of females, working 32 h or more per week, and the highest household income (5th quintile) (Table 3).

**Table 3:** Sociodemographic characteristics of questionnaire respondents and drinkers

	Questionnaire respondents		Drinkers	
	Reference (n = 32 309)	Physicians (n = 1 947)	Reference (n = 29 168; 90.3%)	Physicians (n = 1 759; 90.3%)
Gender *(n (%))*				
Male	15 238 (47.2)	662 (34.0)	14 121 (48.4)	607 (34.5)
Female	17 071 (52.8)	1 285 (66.0)	15 047 (51.6)	1 152 (65.5)
Age in years *(mean (SD))*	41.9 (10.7)	42.1 (10.2)	41.9 (10.8)	42.2 (10.2)
25—34 *(n (%))*	9 865 (30.5)	559 (28.7)	8 877 (30.4)	505 (28.7)
35—44 *(n (%))*	9 924 (30.7)	631 (32.4)	8 874 (30.4)	559 (31.8)
45—54 *(n (%))*	7 593 (23.5)	479 (24.6)	6 884 (23.6)	441 (25.1)
55—65 *(n (%))*	4 927 (15.2)	278 (14.3)	4 533 (15.5)	254 (14.4)
Country of birth *(n (%))*				
The Netherlands	29 381 (90.9)	1 785 (91.7)	26 892 (92.2)	1 629 (92.6)
European	834 (2.6)	37 (1.9)	729 (2.5)	31 (1.8)
Non-European	2 094 (6.5)	125 (6.4)	1 547 (5.3)	99 (5.6)
Specialty group *(n (%))*				
General practice	NA	466 (23.9)	NA	412 (23.4)
(Psycho) social		271 (13.9)		248 (14.1)
Contemplative somatic		415 (21.3)		379 (21.5)
Surgical or supportive		503 (25.8)		265 (15.1)
No specialty		292 (15.0)		455 (25.9)
Educational background *(n (%))*				
Teaching	2 852 (8.8)	NA	2 506 (8.6)	NA
Humanities and arts	3 221 (10.0)		2 845 (9.8)	
Social sciences, business and law	13 878 (43.0)		12 637 (43.3)	
Science, mathematics and computing	2 774 (8.6)		2 459 (8.4)	
Engineering, manufacturing and construction	3 134 (9.7)		2 916 (10.0)	
Agriculture and veterinary	741 (2.3)		673 (2.3)	
Health and welfare (including medicine)	4 548 (14.1)		4 090 (14.0)	
Services	567 (1.8)		514 (1.8)	
Working hours per week *(n (%))*				
None or less than 1	3 089 (9.6)	78 (4.0)	2 607 (8.9)	67 (3.8)
1 to 12	522 (1.6)	16 (0.8)	445 (1.5)	14 (0.8)
12 to 31	5 332 (16.5)	299 (15.4)	4 733 (16.2)	267 (15.2)
32 or more	21 856 (67.6)	1 462 (75.1)	20 459 (70.1)	1 347 (76.6)
Household income *(n (%))*				
1st quintile (lowest income)	2 102 (6.5)	69 (3.5)	1 781 (6.1)	60 (3.4)
2nd quintile	1 663 (5.1)	30 (1.5)	1 380 (4.7)	20 (1.1)
3rd quintile	3 493 (10.8)	89 (4.6)	3 105 (10.6)	78 (4.4)
4th quintile	6 875 (21.3)	272 (14.0)	6 172 (21.2)	243 (13.8)
5th quintile (highest income)	17 812 (55.1)	1 477 (75.9)	16 433 (56.3)	1 350 (76.7)

*n* number, *NA* Not Applicable, *SD* Standard Deviation

### Alcohol consumption

Overall, the vast majority (90.3%) of respondents among physicians and the reference population regularly drank alcohol (Table 3). When looking at the distribution of alcohol consumption patterns within drinkers, physician drinkers complied somewhat more often with the alcohol consumption recommendation (36.5%) and showed somewhat lower proportions of heavy (4.0%) and excessive drinking (4.3%) compared to drinkers in the reference population (32.5%, 7.7%, and 6.4% respectively) (Table 4). Compliance with the alcohol consumption recommendation was overrepresented among drinkers aged 35–54 years, born in European and non-European countries, working less than 32 h per week, and with a household income lower than the 5th quintile. Among heavy and/or excessive drinkers, the specialty groups (psycho) social medicine and no specialty and educational backgrounds social sciences, business and law, services, and humanities and arts were overrepresented.

**Table 4:** Distribution of alcohol consumption patterns^§^ within drinkers by sociodemographic characteristics

	Drinkers		Compliance with alcohol consumption recommendation		Heavy drinkers		Excessive drinkers	
	reference (n = 29 168)	Physicians (n = 1 759)	Reference (n = 9 477; 32.5%)	Physicians (n = 642; 36.5%)	Reference (n = 2 240; 7.7%)	Physicians (n = 70; 4.0%)	Reference (n = 1 864; 6.4%)	Physicians (n = 76; 4.3%)
Gender *(n (%))*								
Male	14 121	607	3 170 (22.4)	147 (24.2)	1 250 (8.9)	21 (3.5)	908 (6.4)	22 (3.6)
Female	15 047	1 152	6 307 (41.9)	495 (43.0)	990 (6.6)	49 (4.3)	956 (6.4)	54 (4.7)
Age in years *(mean (SD))*	41.9 (10.8)	42.2 (10.2)	42.0 (10.2)	42.6 (10.0)	40.3 (12.0)	41.2 (12.3)	46.4 (12.3)	47.3 (11.9)
25–34 *(n (%))*	8 877	505	2 624 (29.6)	163 (32.3)	955 (10.8)	31 (6.1)	434 (4.9)	14 (2.8)
35–44 *(n (%))*	8 874	559	3 218 (36.3)	224 (40.1)	474 (5.3)	11 (2.0)	347 (3.9)	16 (2.9)
45–54 *(n (%))*	6 884	441	2 308 (33.5)	162 (36.7)	430 (6.2)	15 (3.4)	496 (7.2)	21 (4.8)
55–65 *(n (%))*	4 533	254	1 327 (29.3)	93 (36.6)	381 (8.4)	13 (5.1)	587 (12.9)	25 (9.8)
Country of birth *(n (%))*								
The Netherlands	26 892	1 629	8 149 (30.3)	570 (35.0)	2 093 (7.8)	65 (4.0)	1 719 (6.4)	68 (4.2)
European	729	31	307 (42.1)	14 (45.2)	50 (6.9)	*	53 (7.3)	*
Non-European	1 547	99	1 021 (66.0)	58 (58.6)	97 (6.3)	*	92 (5.9)	*
Specialty group *(n (%))*								
General practice	NA	412	NA	153 (37.1)	NA	16 (3.9)	NA	16 (3.9)
(Psycho) social		248		86 (34.7)		14 (5.6)		16 (6.5)
Contemplative somatic		379		154 (40.6)		*		13 (3.4)
Surgical or supportive		265		75 (28.3)		*		11 (4.2)
No specialty		455		174 (38.2)		28 (6.2)		20 (4.4)
Educational background *(n (%))*								
Teaching	2 506	NA	1 032 (41.2)	NA	164 (6.5)	NA	160 (6.4)	NA
Humanities and arts	2 845		1 115 (39.2)		217 (7.6)		239 (8.4)	
Social sciences, business and law	12 637		3 574 (28.3)		1 176 (9.3)		902 (7.1)	
Science, mathematics and computing	2 459		909 (37.0)		161 (6.5)		123 (5.0)	
Engineering, manufacturing and construction	2 916		777 (26.6)		209 (7.2)		149 (5.1)	
Agriculture and veterinary	673		248 (36.8)		26 (3.9)		31 (4.6)	
Health and welfare (including medicine)	4 090		1 459 (35.7)		223 (5.5)		191 (4.7)	
Services	514		172 (33.5)		42 (8.2)		35 (6.8)	
Working hours per week *(n (%))*								
None or less than 1	2 607	67	1 233 (47.3)	35 (52.2)	227 (8.7)	*	284 (10.9)	*
1 to 12	445	14	211 (47.4)	9 (64.3)	30 (6.7)	*	44 (9.9)	*
12 to 31	4 733	267	2 149 (45.4)	125 (46.8)	271 (5.7)	10 (3.7)	271 (5.7)	14 (5.2)
32 or more	20 459	1 347	5 574 (27.2)	457 (33.9)	1 620 (7.9)	52 (3.9)	1 186 (5.8)	51 (3.8)
Household income *(n (%))*								
1st quintile (lowest income)	1 781	60	672 (37.7)	26 (43.3)	265 (14.9)	6 (10.0)	184 (10.3)	*
2nd quintile	1 380	20	617 (44.7)	14 (70.0)	129 (9.3)	*	93 (6.7)	*
3rd quintile	3 105	78	1 164 (37.5)	32 (41.0)	281 (9.0)	*	197 (6.3)	*
4th quintile	6 172	243	2 185 (35.4)	101 (41.6)	397 (6.4)	8 (3.3)	311 (5.0)	*
5th quintile (highest income)	16 433	1 350	4 703 (28.6)	463 (34.3)	1 150 (7.0)	53 (3.9)	1 064 (6.5)	67 (5.0)

*n* number, *NA* Not Applicable, *SD* Standard Deviation

^§^ Row percentages do not add up to 100%, since the alcohol consumption patterns were not mutually exclusive and moderate drinkers were not presented

*small numbers are not reported to prevent disclosure

## Discussion

This study aimed to investigate clinical SUD diagnoses and alcohol consumption patterns among Dutch physicians and a reference population of highly educated Dutch citizens. Using nationwide mental healthcare claims data and health questionnaires, the overall prevalence of clinical SUD diagnoses was low and comparable between physicians and the reference population. Physician SUD patients more often had a sedative use disorder compared to SUD patients in the reference population. Physicians generally had healthier alcohol consumption patterns benchmarked to the reference population. SUD patients and heavy and/or excessive drinking were overrepresented among the specialty group (psycho) social medicine and physicians with no specialty.

Our results showed similar findings for physicians and a comparable reference population with regard to the prevalence of SUD diagnoses and problematic alcohol use. Prevalence rates among physicians seemed (slightly) lower than prevalence rates in the reference population, which might be explained by differences in sociodemographic characteristics. For example, females showed lower rates of SUD diagnoses and problematic alcohol use and they were better represented among physicians than among the reference population. Additionally, with regard to an overall higher income among physicians, this seems to be associated with less problematic alcohol use. Overall, we observed relatively low prevalence rates of SUD diagnoses (0.3% and 0.5% respectively) and problematic alcohol use (3.6% and 6.9% heavy drinkers and 3.9% and 5.8% excessive drinkers respectively) in both physicians and the reference population, compared to the general population that meets the criteria for SUD diagnosis (alcohol use disorder 5.1% and drug use disorder 0.7%) and problematic alcohol use (18.2%) worldwide [35, 40]. This lower prevalence of substance use related issues in our sample might be explained by protective effects for the development of SUD of high SES in our sample, a lower willingness to seek help (larger treatment gap) among highly educated citizens, or by the assessment procedure since only claims registrations were included. Given the voluntary nature of the Dutch PHP and the fact that Dutch physicians with SUD are hardly reported to the Dutch regulator, we do not expect regulatory issues to have led to underreporting or low numbers of treatment seeking. These findings however do not support the suggestion that physicians are at increased risk for problematic alcohol use [16, 26, 36, 41], which is beneficial for physicians as well as for the quality of care and patient safety.

Benchmarked to reference patients, physician patients were more often diagnosed with a SUD on sedative, hypnotic, or anxiolytic substances (like benzodiazepines) and less often with a SUD on alcohol. This is largely in line with studies among physicians in Australia and the United States, showing that a significant part of SUD diagnoses among physicians was related to other substance(s) than alcohol, including prescription drugs [1, 27, 39]. It has been suggested that this might be the consequence of physicians’ authority to prescribe drugs, which makes physicians more familiar with and gives them easier access to prescription drugs [14]. A cross-sectional survey among 729 young Irish physicians found that 3–7% of the respondents had prescribed themselves benzodiazepines, opioids, or other psychotropic medication [14]. Male physicians and physicians with a surgical or supportive specialty were at higher risk of self-prescribing addictive medication [14]. As previously found by a review about self-medication in physicians and medical students, physicians continue to self-prescribe medication despite clear professional guidelines [20], including addictive drugs [14].

Since a higher proportion of physician SUD patients experienced mild to no symptoms at the end of treatment compared to reference SUD patients, this might indicate a better prognosis for physician SUD patients than for reference SUD patients. This is consistent with our recent meta-analysis showing that healthcare professionals who participated in a monitoring program were about 1.5 times more likely to achieve long-term abstinence compared to general relapse rates of 50% in the first year after treatment, [13, 18, 19, 21, 38]. This better prognosis might not only be explained by participation in the monitoring program, but also by protective and supportive socioeconomic factors (like educational level, income, and occupation).

In this study, the specialty groups psycho (social) medicine and physicians with no specialty were associated with higher proportions of SUD diagnoses and/or heavy and excessive drinking. This observation may have several explanations, such as an actually higher SUD rate, a higher rate of SUD identification, and/or a higher rate of help seeking. It can also be speculated that development of an SUD hinders specialist training, resulting in physicians with no specialty being overrepresented among the SUD group, and showing higher alcohol consumption levels. Future studies should further investigate whether certain physicians are more at risk for substance use related problems than others, and why this is the case.

Strengths of the current study include the use of nationwide data with large sample sizes and the use of a highly educated reference population enabling decent comparison of the prevalence of SUD diagnoses and alcohol consumption patterns among physicians. Since we presented nationwide data of the Netherlands, our findings may not be generalizable to other countries. The reference population in our benchmark was selected based on a high educational level (Master or PhD degree), ideally this selection was also based on a high income, to reflect the high socioeconomic status of physicians. Unfortunately, Statistics Netherlands had no income data available in the demographics register on a nationwide level, so therefore we were not able to take that into account. We did not present a group of moderate drinkers, which is not a problem since heavy and excessive drinkers were our main interest. A potential limitation of this study is that some characteristics within SUD patients and within alcohol consumption patterns had relatively small numbers, which may lead to a higher level of uncertainty in the observed prevalence rates. Moreover, prevalence rates of clinical SUD diagnoses might be underestimated due to a treatment gap and prevalence rates of heavy and/or excessive drinking might be underestimated due to social desirable answers. It remains to be studied whether this affects prevalence rates more in physicians than in the general population.

## Conclusions

This is the first study that investigated prevalence rates of clinical SUD diagnoses and alcohol consumption patterns among physicians using nationwide data and a highly educated reference population. Prevalence rates of clinical SUD diagnoses and alcohol consumption patterns were fairly comparable or slightly more favorable among physicians compared to the reference population. Despite the relatively low levels of SUD and heavy and/or excessive alcohol consumption, substance use related problems among physicians remain an important topic from a healthcare perspective. Special attention should be directed to the use of sedatives, since physician SUD patients were more often diagnosed with a sedative use disorder than non-physician SUD patients.

## Supplementary Information

**Additional file 1: Table S1. **Distribution of various medical specialties. **Table S2. **Definitions of substance of abuse or dependence and comorbid psychiatric disorders by DSM-IV codes. **Table S3. **Sociodemographic characteristics of questionnaire respondents.

## Data Availability

The data that support the findings of this study are available from Statistics Netherlands but restrictions apply to the availability of these data, which were used under license for the current study and are not publicly available.
