# Supplementary material for: Substance use disorder and alcohol consumption patterns among Dutch physicians: a nationwide register-based study
Source: Addict Sci Clin Pract. 2023 Jan 13;18:4. doi: 10.1186/s13722-022-00356-9 (PMC9837897; doi:10.1186/s13722-022-00356-9)
Supplement: Supplementary file 1 — Additional file 1: Table S1. Distribution of various medical specialties. Table S2. Definitions of substance of abuse or dependence and comorbid psychiatric disorders by DSM-IV codes. Table S3. Sociodemographic characteristics of questionnaire respondents. [file 13722_2022_356_MOESM1_ESM.docx]

**Additional file 1**

**Table S1.** Distribution of various medical specialties

| **Specialty group** | | | | |
| --- | --- | --- | --- | --- |
| General practice | (Psycho) social | Contemplative somatic | Supportive and surgical | No specialty |
| general practice | addiction medicine | allergology | anesthesiology | no specialty |
|  | forensic medicine | cardiology | cardiothoracic surgery |  |
|  | infectious disease control | dermatology and venereology | clinical chemistry |  |
|  | insurance medicine | internal medicine | clinical genetics |  |
|  | mental disability | gastroenterology | general surgery |  |
|  | occupational medicine | geriatrics | medical microbiology |  |
|  | policy and advice | neurology | neurosurgery |  |
|  | psychiatry | pediatrics | nuclear medicine |  |
|  | public health | pneumology | obstetrics and gynecology |  |
|  | social medical assessment and counseling | rehabilitation medicine | ophthalmology |  |
|  | tuberculosis control | rheumatology | orthopedics |  |
|  | youth health care | sports medicine | otolaryngology |  |
|  |  |  | pathology |  |
|  |  |  | plastic surgery |  |
|  |  |  | emergency medicine |  |
|  |  |  | radiology |  |
|  |  |  | radiotherapy |  |
|  |  |  | urology |  |

**Table S2.** Definitions of substance of abuse or dependence and comorbid psychiatric disorders by DSM-IV codes

|  | | DSM-IV codes |
| --- | --- | --- |
| Substance of abuse or dependence | |  |
|  | Alcohol | 303.9x and 305.0x |
|  | Amphetamine | 304.4x and 305.7x |
|  | Cannabis | 304.3x and 305.2x |
|  | Cocaine | 304.2x and 305.6x |
|  | Opioid | 304.0x and 305.5x |
|  | Sedative, hypnotic, or anxiolytic | 304.1x and 305.4x |
|  | Other or unknown substance(s) | 304.5x, 304.6x, 304.8x, 304.9x, 305.3x, 305.9x |
| Comorbid psychiatric disorder | |  |
|  | Developmental disorder | 299.x, 307.0, 307.2x, 307.3, 307.5x, 307.6, 307.7, 307.9, 309.21, 312.8, 312.9, 313.2x, 313.81, 313.89, 313.9, 314.x, 315.x, 317, 318.x, 319 |
|  | Cognitive disorder | 293.0, 290.x, 294.x, 780.0x |
|  | Psychotic disorder | 293.81, 293.82, 295.x, 297.x, 298.x |
|  | Mood disorder | 293.83, 296.x, 300.4, 301.1x, 311 |
|  | Anxiety disorder | 293.84, 293.89, 300.0x, 300.2x, 300.3, 308.3, 309.8x |
|  | Somatoform and/or dissociative disorder | 300.11, 300.2x, 300.3, 300.4, 300.6, 300.7, 300.8x, 300.12, 300.14, 300.15, 307.8x |
|  | Personality disorder | 301.0, 301.2x, 301.4, 301.5x, 301.6, 301.7, 301.8x, 301.9 |
|  | Other psychiatric disorder | mental disorders due to a general medical condition, factitious disorders, sexual and gender identity disorders, eating disorders, sleep disorders, impulse-control disorders not else classified, and adjustment disorders |

**Table S3.** Sociodemographic characteristics of questionnaire respondents

|  | | Public Health monitor respondents (2012 + 2016) | |  | Health survey respondents  (2014 - 2019) | |  | Questionnaire respondents (monitor + survey) | |
| --- | --- | --- | --- | --- | --- | --- | --- | --- | --- |
|  | | reference (n = 29 597) | physicians  (n = 1 808) |  | reference (n = 2 712) | physicians  (n = 139) |  | reference (n = 32 309) | physicians  (n = 1 947) |
| Gender *(n (%))* | |  |  |  |  |  |  |  |  |
|  | Male | 13 926 (47.1) | 612 (33.8) |  | 1 312 (48.4) | 50 (36.0) |  | 15 238 (47.2) | 662 (34.0) |
|  | Female | 15 671 (52.9) | 1 196 (66.2) |  | 1 400 (51.6) | 89 (64.0) |  | 17 071 (52.8) | 1285 (66.0) |
| Age in years *(mean (SD))* | | 41.8 (10.7) | 42.2 (10.3) |  | 41.5 (10.6) | 40.9 (9.0) |  | 41.9 (10.7) | 42.1 (10.2) |
|  | 25 – 34 *(n (%))* | 9 019 (30.5) | 516 (28.5) |  | 846 (31.2) | 43 (30.9) |  | 9 865 (30.5) | 559 (28.7) |
|  | 35 – 44 *(n (%))* | 9 115 (30.8) | 582 (32.2) |  | 809 (29.8) | 49 (35.3) |  | 9 924 (30.7) | 631 (32.4) |
|  | 45 – 54 *(n (%))* | 6 910 (23.3) | 444 (24.6) |  | 683 (25.2) | 35 (25.2) |  | 7 593 (23.5) | 479 (24.6) |
|  | 55 – 65 *(n (%))* | 4 553 (15.4) | 266 (14.7) |  | 374 (13.8) | 12 (8.6) |  | 4 927 (15.2) | 278 (14.3) |
| Country of birth *(n (%))* | |  |  |  |  |  |  |  |  |
|  | The Netherlands | 26 962 (91.1) | 1 660 (91.8) |  | 2 419 (89.2) | 125 (89.9) |  | 29 381 (90.9) | 1 785 (91.7) |
|  | European | 761 (2.6) | 36 (2.0) |  | 73 (2.7) | * |  | 834 (2.6) | 37 (1.9) |
|  | Non-European | 1874 (6.3) | 112 (6.2) |  | 220 (8.1) | * |  | 2094 (6.5) | 125 (6.4) |
| Specialty group *(n (%))* | |  |  |  |  |  |  |  |  |
|  | General practice | NA | 432 (23.9) |  | NA | 34 (24.5) |  | NA | 466 (23.9) |
|  | (Psycho) social |  | 250 (13.8) |  |  | 21 (15.1) |  |  | 271 (13.9) |
|  | Contemplative somatic |  | 383 (21.2) |  |  | 32 (23.0) |  |  | 415 (21.3) |
|  | Surgical and supportive |  | 274 (15.2) |  |  | 18 (12.9) |  |  | 503 (25.8) |
|  | None |  | 469 (25.9) |  |  | 34 (24.5) |  |  | 292 (15.0) |
| Educational background *(n (%))* | |  |  |  |  |  |  |  |  |
|  | Teaching | 2 617 (8.8) | NA |  | 235 (8.7) | NA |  | 2 852 (8.8) | NA |
|  | Humanities and arts | 2 956 (10.0) |  |  | 265 (9.8) |  |  | 3 221 (10.0) |  |
|  | Social sciences, business and law | 12 656 (42.8) |  |  | 1 222 (45.1) |  |  | 13 878 (43.0) |  |
|  | Science, mathematics and computing | 2 556 (8.6) |  |  | 218 (8.0) |  |  | 2 774 (8.6) |  |
|  | Engineering, manufacturing and construction | 2 899 (9.8) |  |  | 235 (8.7) |  |  | 3 134 (9.7) |  |
|  | Agriculture and veterinary | 693 (2.3) |  |  | 48 (1.8) |  |  | 741 (2.3) |  |
|  | Health and welfare (including medicine) | 4 209 (14.2) |  |  | 339 (12.5) |  |  | 4 5848 (14.1) |  |
|  | Services | 516 (1.7) |  |  | 51 (1.9) |  |  | 567 (1.8) |  |
| Working hours per week *(n (%))* | |  |  |  |  |  |  |  |  |
|  | Not working or less than 1 | 2 859 (9.7) | 78 (4.3) |  | 230 (8.5) | 0 (0.0) |  | 3 089 (9.6) | 78 (4.0) |
|  | 1 to 12 | 468 (1.6) | 15 (0.8) |  | 54 (2.0) | 1 (0.7) |  | 522 (1.6) | 16 (0.8) |
|  | 12 to 31 | 4 880 (16.5) | 275 (15.2) |  | 452 (16.7) | 24 (17.3) |  | 5 332 (16.5) | 299 (15.4) |
|  | 32 or more | 19 880 (67.2) | 1 348 (74.6) |  | 1 976 (72.9) | 114 (82.0) |  | 21 856 (67.6) | 1 462 (75.1) |
| Household income *(n (%))* | |  |  |  |  |  |  |  |  |
|  | 1^st^ quintile (lowest income) | 1 921 (6.5) | 67 (3.7) |  | 181 (6.7) | 2 (1.4) |  | 2 102 (6.5) | 69 (3.5) |
|  | 2^nd^ quintile | 1 505 (5.1) | 29 (1.6) |  | 158 (5.8) | 1 (0.7) |  | 1 663 (5.1) | 30 (1.5) |
|  | 3^rd^ quintile | 3 180 (10.7) | 82 (4.5) |  | 320 (11.5) | 7 (5.0) |  | 3 493 (10.8) | 89 (4.6) |
|  | 4^th^ quintile | 6 285 (21.2) | 247 (13.7) |  | 609 (21.8) | 25 (18.0) |  | 6 875 (21.3) | 272 (14.0) |
|  | 5^th^ quintile (highest income) | 16 371 (55.3) | 1 373 (75.9) |  | 1 483 (53.2) | 104 (74.8) |  | 17 812 (55.1) | 1 477 (75.9) |

n = number, NA = Not Applicable, SD = Standard Deviation, * small numbers are not reported to prevent disclosure.
